# Real-time Feedback to Improve HIV Treatment Adherence in Pregnant and Postpartum Women in Uganda: A Randomized Controlled Trial

**DOI:** 10.1007/s10461-022-03712-7

**Published:** 2022-06-15

**Authors:** Lora L. Sabin, Elizabeth Simmons, Nafisa Halim, Davidson H. Hamer, Allen L. Gifford, Rebecca L. West, Anna Larson, Rachael Bonawitz, Philip Aroda, Bolanle Banigbe, Alayna J. Holderman, Lisa Murray, Mary B. DeSilva, Julia Gasuza, Barbara Mukasa, Lisa J. Messersmith

**Affiliations:** 1 Department of Global Health, Boston University School of Public Health, 801 Massachusetts Avenue, Crosstown Center, 3rd Floor, 02118 Boston, MA USA; 2 Carolina Population Center, University of North Carolina at Chapel Hill, 27516 Chapel Hill, NC USA; 3 Department of Medicine, Boston University School of Medicine, 02118 Boston, MA USA; 4 Department of Health Law, Policy, and Management, Boston University School of Public Health, 02118 Boston, MA USA; 5 Center for Healthcare Organization and Implementation Research, VA Boston Healthcare System, 02130 Boston, MA United States; 6 Mildmay Uganda, Kampala Road, Kampala, Uganda; 7 Department of Epidemiology & Biostatistics, Boston University School of Public Health, 02118 Boston, MA USA; 8 Department of Human Genetics, University of Pittsburgh School of Public Health, 15261 Pittsburgh, PA USA; 9 Center for Excellence in Public Health, University of New England, 04103 Portland, ME USA

**Keywords:** Pregnant and postpartum women, HIV treatment adherence, Randomized controlled trial, Uganda

## Abstract

We assessed an intervention aimed at improving adherence to antiretroviral therapy (ART) among pregnant and postpartum women living with HIV (PPWLH). We randomized 133 pregnant women initiating ART in Uganda to receive text reminders generated by real time-enabled electronic monitors and data-informed counseling through 3 months postpartum (PPM3) or standard care. Intention-to-treat analyses found low adherence levels and no intervention impact. Proportions achieving ≥95% adherence in PPM3 were 16.4% vs. 9.1% (t = -1.14, p *=* 0.26) in intervention vs. comparison groups, respectively; 30.9% vs. 29.1% achieved ≥80% adherence. Additional analyses found significant adherence declines after delivery, and no effect on disease progression (CD4-cell count, viral load), though treatment interruptions were significantly fewer in intervention participants. Per-protocol analyses encompassing participants who used adherence monitors as designed experienced better outcomes, suggesting potential benefit for some PPWLH. The study was registered on ClinicalTrials.Gov (NCT02396394).

**Supplementary Information:**

The online version contains supplementary material available at 10.1007/s10461-022-03712-7.

## Introduction

Antiretroviral therapy (ART) remains the only effective treatment for people living with HIV (PLWH). Due to major efforts globally to expand access to ART, most recently reflected in the latest recommendations of the World Health Organization (WHO) to “test and treat,” the number of PLWH on treatment continues to grow rapidly [1]. However, medication adherence is an ongoing challenge as ART provision expands [2]. Although what constitutes optimal adherence is controversial, it typically ranges from 80 to 95% of prescribed doses [3–5], above the 60–80% adherence levels often observed [3, 6–8]. Additionally, adherence may change over a lifetime of ART use and is subject to disruptions in treatment routines [2, 9]. Sustained treatment interruptions increase both the odds of viral rebound [10] and the likelihood of drug resistance [11], making such interruptions a crucial and nuanced component of adherence measurement.

Research has shown that barriers to adherence vary across individuals and settings [9, 12]. Multiple adherence enhancement interventions have been assessed, with varying levels of evidence for their effectiveness and potential for scalability [2, 13]. Mobile health (mHealth) approaches have shown exciting potential for promoting adherence, including wireless, real-time monitoring strategies that permit early identification of adherence lapses and immediate intervention to provide support. Use of real-time wireless pill monitors (WPM) is feasible and acceptable in resource-limited settings in a wide range of populations [5, 14–19], although the evidence is mixed regarding the effect of interventions utilizing WPM [10, 20–22]. In one randomized controlled trial (RCT) conducted by members of our team, triggered reminders combined with data-informed counseling increased mean adherence and proportion achieving ≥ 95% adherence among Chinese patients [20]. A similar South African trial found no evidence that triggered reminders improved adherence, but they did reduce treatment interruptions [21]. Another study in Uganda observed a significant increase in mean ART adherence after switching a patient cohort from standard electronic adherence monitoring to WPM, accompanied by follow-up visits for patients with sustained interruptions [10]. Participants receiving real-time adherence monitoring with SMS reminders in both China and Uganda reported feeling ‘seen’ and ‘cared about,’ motivating them to improve adherence [23, 24].

Pregnant women are a priority group within the global HIV response as they represent an opportunity to reduce risk of vertical transmission, in addition to the individual benefits of ART. They are also known to experience relatively poor ART adherence and retention in care [25–28]. Retention rates in pregnant and postpartum women living with HIV (PPWLH) in sub-Saharan Africa (72.9–76.4%) are below those of the general adult population [25], even in the era of Option B+, whereby all pregnant women who test positive for HIV are offered ART immediately [29]. Poor ART adherence is associated with fear of HIV disclosure [28, 30–32], HIV-related stigma [28, 30, 32, 33], lack of self-efficacy [34], and weak social and family support [32, 35]. Given these challenges and evidence of improved adherence associated with use of real-time adherence monitoring in other populations, we conducted an RCT to determine the efficacy of real-time feedback plus data-informed counseling on ART adherence among PPWLH in Uganda.

## Methods

### Setting

This study was conducted in central Uganda. In 2020, 1.4 million people in Uganda were living with HIV, with higher prevalence among women (6.9% of adult women compared to 5.3% of adult men) [36]. Uganda was among the first countries in sub-Saharan Africa to adopt Option B+; as of March 2014, Option B + had been implemented in all antenatal (ANC) care facilities in Uganda as standard of care [37, 38]. This RCT enrolled ART-naïve PPWLH at two high-volume government-operated clinics, Mityana Hospital in Mityana district and Entebbe Grade B Hospital in Wakiso district. Both clinics provide integrated ANC and ART services for PPWLH. More details on standard of care, study sites, and retention outcomes were reported previously [17].

### Study participants and pre-intervention period

Individuals were eligible for the study if they were pregnant, between 12 and 26 weeks of gestation, 18 years of age or older, receiving integrated ANC/ART services at one of the study hospitals, and initiating ART for the first time. Participants were required to use a cell phone that received text messages in their homes, and were provided with a phone if they did not own one. All participants provided written informed consent before enrollment. Participants received the equivalent of $4 at the end of each clinic visit as reimbursement for travel costs and lost work associated with study participation.

Upon enrollment, the onsite study coordinator at each hospital provided each participant with a WPM (Wisepill Technologies, Cape Town, South Africa) [39] and instructions on its use. Every participant’s WPM was then monitored daily for a one-month pre-intervention period to confirm usage. The WPM recorded the date and time of each opening and transmitted these data immediately to a central server. When a participant experienced a two-day period without WPM openings, a study coordinator contacted them to ascertain the reason for non-use. In the event a participant had poor reception or indicated intention to discontinue use of the device, they were withdrawn from the study. Participants who did not attend the regularly scheduled one-month clinic visit within a four-week grace period were also withdrawn.

### Randomization

At the one-month visit, participants continuing in the study (confirmed by ability to use the WPM as intended and to attend their next scheduled hospital visit) were assigned to a study arm using block randomization. A study researcher using an electronic randomization tool assigned participants 1:1 to intervention vs. comparison arm within blocks of 10, and then conveyed assignments to study coordinators in Uganda via a secure transfer system.

### Intervention overview

Following randomization, intervention participants selected a text reminder to receive on their cell phones if the WPM was unopened within two hours of the prescribed dose time. The text messages were designed to be friendly, non-stigmatizing, and not harmful (for example, using language disclosing the woman’s HIV status). Examples of messages participants could choose included “Time for prayers” or “Hello, it’s time.” In addition to the reminders, intervention participants were eligible to receive WPM data-informed adherence and retention counseling at monthly clinic visits. At each visit, intervention participants received a report generated by the WPM depicting a calendar view of doses taken by day and time in the previous month, and a summary of doses taken on time. Successful dose adherence was defined as taking the dose within a two-hour period of the pre-determined dose time chosen by the participant. Intervention participants with < 95% adherence in the previous month participated in a report-informed counseling session with a trained clinic counselor in a private room at the clinic; for intervention participants with higher adherence, report-informed counseling was encouraged but optional. Counseling sessions focused on assessing the reasons for any lapses in adherence and strategies for improvement in the coming month. Adherence counseling sessions were not pre-scripted to allow for spontaneous discussions, but all counsellors underwent training regarding the use of the report, as well as supportive (rather than punitive) counseling. At month 3 postpartum (PPM3), participants stopped receiving the reminders and data-informed counseling.

### Comparison arm procedures

The comparison arm received all standard services for both antenatal and postpartum care, and standard of care ART support, including regular counseling, during the intervention period. They used the WPM device throughout the study but did not receive reminder SMS messages, WPM-generated adherence reports, or data-informed counseling.

Due to the nature of the intervention, neither participants nor counselors could be blinded to randomization assignment. Extensive training provided by study clinicians prior to beginning the trial aimed to ensure consistent patient support among counselors at the clinic sites. Training activities stressed the importance of patient attendance at every scheduled monthly visit, taking medications at the right time each day, providing comprehensive and non-judgmental counseling, and practicing with WPM reports.

Given enrollment at varied times of gestation (12–26 weeks) and the unpredictability of delivery, potential trial participation ranged from one to six months pre-delivery in addition to three months post-delivery. Throughout the intervention period, the study team attempted to contact participants (regardless of randomization assignment) who missed a scheduled visit to remind them of their missed appointment and to urge them to collect their medications at the hospital. For the last intervention visit at PPM3, study staff recorded whether the participant attended the visit and completed the intervention; if a participant missed the PPM3 visit but completed a visit in the subsequent three months, they were designated as having completed the intervention.

### Outcomes

The primary outcome was the proportion of participants achieving ≥ 95% adherence during the final 30 days of the intervention period. Secondary adherence outcomes encompassed ≥ 95% adherence over the entire intervention period and pre- and post-delivery periods separately. We also assessed proportions achieving a ≥ 80% adherence threshold and mean adherence over the same time periods and explored adherence patterns using mean monthly adherence over the entire intervention period. For adherence outcomes, detailed records of all participants’ adherence were constructed using date and time records of each device opening throughout the study period. Adherence was defined as: (number of doses taken ± 2 h of dose time)/(total number of prescribed doses) with doses taken outside the ± 2-hour window considered non-adherent, a time-sensitive adherence measure used in other studies [16, 20, 40]. For days with multiple device openings, a participant was considered adherent if at least one opening occurred during the ±2-hour window. Days with no device openings were categorized as (a) missing due to behavior (e.g., intentional non-opening, forgetfulness) or (b) missing due to likely device failure, according to the participant’s detailed adherence record, which indicated periods of dead battery or loss of connectivity. Missing data due to likely device failures were excluded from analyses.

In addition to adherence, we calculated the number of treatment interruptions (defined as missing three or more doses in a row) and assessed three measures of HIV disease progression, including mean CD4-cell count, change in CD4-cell count, and viral load suppression at PPM3. CD4-cell count and plasma HIV viral load tests were conducted at enrollment and PPM3. CD4-cell counts were measured as cells/µl using a FACSCount^rTM^ (Becton Dickinson), and were performed at each hospital. Viral load tests were performed using a nucleic acid sequence-based amplification method with Organon Teknica NucliSENS machine (Boxtel, Netherlands), with a lower limit of detection of 50 copies/mL of HIV plasma. Assays were done at the Mildmay Hospital facility in Kampala, which provided technical and laboratory support to both hospitals during the study.

### Sample size and statistical power

The study was powered to detect a 25%-point difference in the proportion achieving ≥ 95% adherence. From previous studies, we estimated, conservatively, that 50% would achieve this threshold [20, 25, 40, 41]. Assuming a minimum of 80% power and a two-sided alpha of 0.05, we estimated that a sample size of 160 pregnant participants (80 per study arm), allowing for 25% attrition, would provide an adequate sample size to detect a difference of 75% vs. 50% in proportions achieving the ≥ 95% adherence threshold.

### Analytic approach

We conducted a primary intention-to-treat (ITT) analysis and a secondary per protocol (PP) analysis for assessments of adherence, treatment interruptions, and markers of HIV disease progression. SAS version 9.4 was used for all primary and secondary analyses. The ITT analysis included data for all randomized participants, though excluded missing adherence data due to WPM device failure as explained above. The PP analysis included only randomized participants who complied with intervention protocol procedures, defined by (a) consistent use of the WPM device (operationalized as having < 10% of missing adherence data, mainly due to device failure resulting from dead batteries) and (b) completion of the intervention, as described above. For missing CD4-cell count and viral load measures at PPM3, both ITT and PP analyses assumed no change between baseline and PPM3, and imputed baseline values.

The primary and secondary adherence outcomes for intervention and comparison participants were compared in each time period using chi-squared tests of independence for categorical variables and two-sample t-tests for continuous variables. Treatment interruptions were compared using a Poisson regression to calculate incidence rate ratios. The model was adjusted by maternal age, gestational age, parity, and study site. Measures of HIV disease progression were compared using chi-squared tests of independence for viral load counts and two-sample t-tests for CD4 counts. *P*-values of less than α = 0.05 were considered significant.

For the adherence patterns analysis, we calculated mean monthly adherence of the PP sample in pre- and post-delivery periods and grouped participants into ‘high’ or ‘low’ adherence in each period, using a ≥ 80% threshold. We then stratified participants into one of four patterns based on pre-/post-delivery adherence: high/high, high/low, low/high, and low/low. To visualize patterns, we plotted mean monthly adherence for each group, by intervention vs. comparison arm.

### Ethical approvals

The study was approved by the research ethics committee of the School of Medicine (SOMREC) at Makarere University’s College of Health Sciences in Kampala, Uganda, the Uganda National Council of Science and Technology (UNCST), and the institutional review board at Boston University. The study was registered on ClinicalTrials.Gov (NCT02396394).

## Results

### Participant characteristics

A total of 165 HIV-positive pregnant women initiating ART were enrolled between June 2015 and January 2016, with equal representation across sites. Approximately 30% of enrolled participants were provided with a cell phone for use during the study. At month one, 133 were eligible to continue in the study (66 in Entebbe and 67 in Mityana) and were randomized to intervention (n = 69) or comparison arm (n = 64). Descriptive analyses of characteristics of those randomized compared to those who were excluded (n = 32) revealed no significant differences [35]. A total of 108 completed the trial; we included 131 participants in the ITT analyses (68 intervention, 63 comparison) (two participants, one in each arm, were withdrawn immediately after randomization and were excluded from all analyses). Overall, a little more than 20% of adherence data was missing due to device failure (mainly from dead batteries and loss of connectivity); mean missing data was 23.6% and 21.4% among intervention and comparison participants, respectively (t = 0.54, p = 0.59). A total of 44 intervention and 34 comparison participants either did not complete the intervention period and/or had > 10% missing data. Thus, 53 individuals met the criteria for inclusion in the PP analysis, 24 intervention and 29 comparison participants. A detailed study flow figure was published previously [17].

Sociodemographic characteristics were similar across arms in both the ITT and PP analyses (Table 1). In the ITT group, the mean age was 25.4 years at randomization; mean gestational age at enrollment was 21 weeks. Nearly three-quarters (74.0%) were married, while 55.0% had completed secondary school or higher. About one-third were pregnant for the first time; the mean number of previous live births was 1.9. Someone else (including partners) knew about the HIV status of 42.0% of participants prior to enrollment; 25.2% disclosed to their husbands or partners at enrollment. Participants included in the PP analysis were similar to the full sample in nearly all respects, although a higher proportion had completed secondary education (63.5% vs. 55.0%), and mean adherence was slightly higher in the pre-intervention period (83.0% vs. 77.9%). There were no significant differences across intervention and comparison arms.

**Table 1:** Sociodemographic characteristics at randomization

	*Intention to treat*								*Per protocol*							
	**Overall** **(n = 131)**		**Intervention** **(n = 68)**		**Comparison** **(n = 63)**		***t-test***	***p***	**Overall** **(n = 53)**		**Intervention** **(n = 24)**		**Comparison** **(n = 29)**		***t-test***	***P***
	**Mean/%**	**(SD)**	**Mean/%**	**(SD)**	**Mean/%**	**(SD)**			**Mean/%**	**(SD)**	**Mean/%**	**(SD)**	**Mean/%**	**(SD)**		
Age (years)^a,b^	25.4	(6.2)	25.7	(4.8)	24.9	(7.5)	-0.68	0.50	26.1	(6.3)	27.1	(4.6)	25.1	(8.0)	-0.97	0.34
Gestation age at enrollment (weeks)^a,b^	21.0	(4.7)	20.3	(5.1)	21.8	(4.3)	1.90	0.06	21.8	(4.4)	21.5	(4.8)	22.0	(4.1)	0.43	0.67
Married^b^	74.0%	(44.0)	70.6%	(45.9)	77.8%	(41.9)	0.93	0.35	76.0%	(43.1)	82.6%	(38.8)	72.4%	(45.5)	-0.86	0.40
Education level completed																
Primary or lower	45.8%	(50.0)	47.1%	(50.3)	44.4%	(50.1)	-0.30	0.76	36.5%	(48.6)	30.4%	(47.1)	41.4%	(50.1)	-0.80	0.43
Secondary or higher^a,b^	55.0%	(49.9)	53.7%	(50.2)	56.5%	(50.0)	0.31		63.5%	(48.6)	70.0%	(47.1)	58.6%	(39.6)	0.80	
First Pregnancy^a,b^	29.0%	(45.6)	25.4%	(43.9)	32.8%	(47.3)	0.90	0.37	26.5%	(44.6)	22.7%	(42.9)	29.6%	(46.5)	0.53	0.60
Number of live births^a,b^	1.9	(1.9)	1.8	(1.6)	2.0	(2.2)	0.35	0.72	2.0	(1.8)	2.0	(1.7)	2.0	(1.8)	0.00	1.00
Someone else knew status at enrollment^b^	42.0%	(49.5)	44.1%	(50.0)	39.7%	(49.3)	-0.51	0.61	46.2%	(50.3)	34.8%	(48.7)	55.2%	(50.6)	1.47	0.15
Disclosed to husband/ partner at enrollment	25.2%	(43.6)	30.9%	(46.5)	19.1%	(39.6)	-1.56	0.12	28.8%	(45.7)	26.1%	(44.9)	31.0%	(47.1)	0.38	0.70
Mean adherence, pre-intervention period^c^	77.9%	(23.3)	79.7%	(22.0)	75.9%	(24.7)	-0.93	0.35	83.0%	(24.4)	86.8%	(22.2)	80.0%	(26.1)	-1.01	0.32

^a^Intention to treat: n is 109 for age (years); 129 for gestation age at enrollment (weeks); 129 for secondary level of education or higher; 124 for first pregnancy; 113 for number of live births

^b^Per protocol: n is 43 for age (years); 52 for gestation age at enrollment (weeks); 52 for married; 52 for secondary level of education or higher; 50 for first pregnancy; 47 for number of live births; and 52 for someone else knew status at enrollment

^c^Adherence is defined as the number of doses taken within 2 h of dose time divided by the total number of prescribed doses

### Effect of real-time feedback on adherence outcomes: ITT analysis

#### ≥95% adherence

**Fig. 1:**
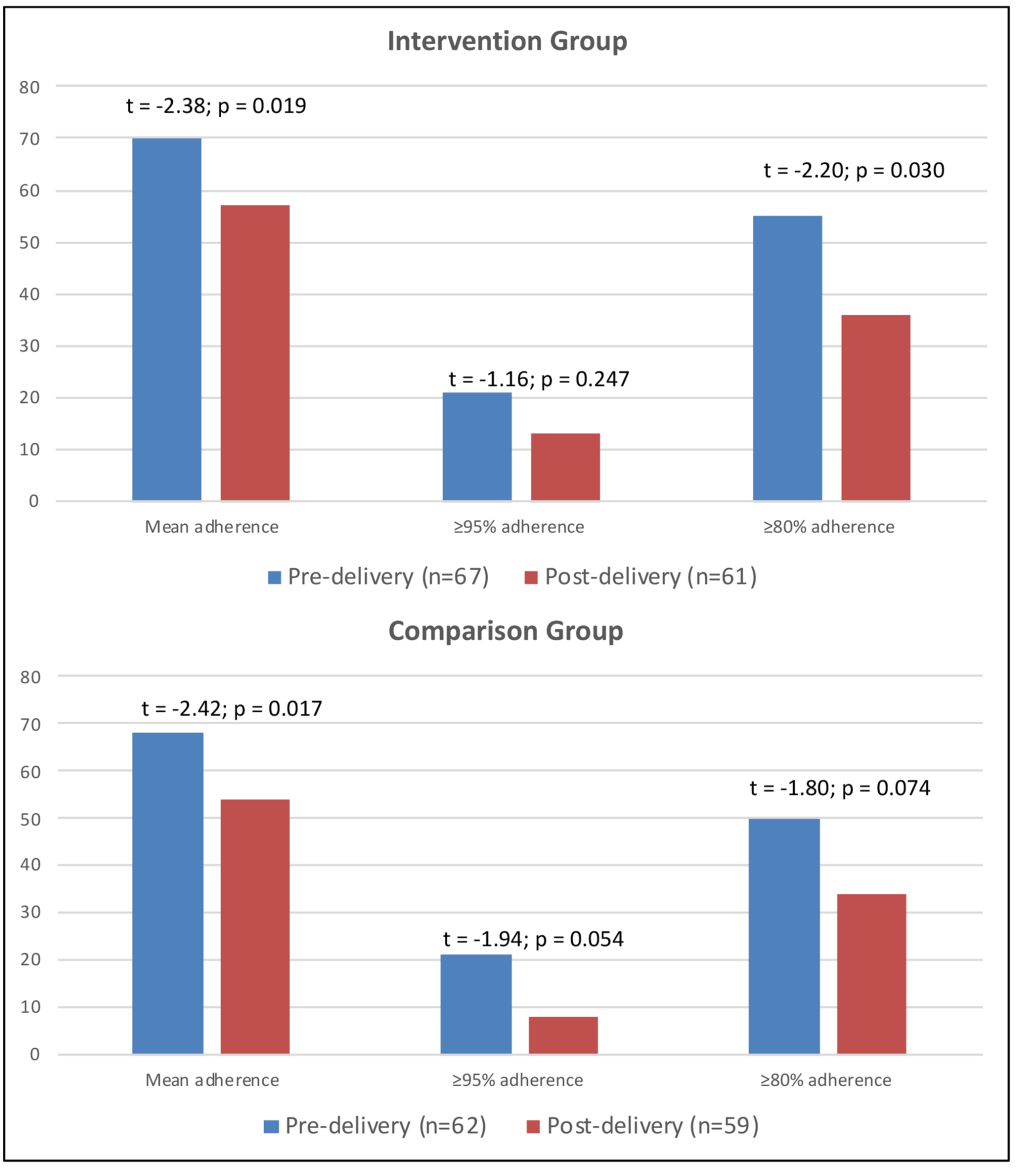
Comparison of pre- and post-delivery adherence outcomes (ITT analysis)

In the final 30 days of the intervention, 16.4% of intervention participants achieved ≥95% adherence, compared to 9.1% in the comparison group (t = -1.14, p *=* 0.26) (Table 2). Across the full intervention period, 11.8% vs. 9.5% (t = -0.41, p = 0.68) of intervention vs. comparison participants, respectively, achieved ≥95% adherence. Pre-delivery, approximately 20% in both groups achieved this threshold (20.9% vs. 21.0%, t = 0.01, p = 0.99), although these proportions fell sharply post-delivery to 13.1% and 8.5% (t = -0.82, p = 0.42), respectively (see also Fig. 1).

**Table 2:** ITT and PP analysis: Primary and secondary study outcomes

	Intention to treat				Per protocol			
	**Intervention** **(n = 68)**	**Comparison** **(n = 63)**	***t-test***	***p***	**Intervention** **(n = 24)**	**Comparison** **(n = 29)**	***t-test***	***p***
**≥ 95% Adherence**, % (95% CI)								
Final 30 days of intervention	16.4 (6.3, 26.5)	9.1 (1.3, 16.9)	-1.14	0.26	16.7 (0.6, 32.7)	10.3 (-1.4, 22.1)	-0.67	0.51
Full Intervention^a^	11.8 (3.9, 19.6)	9.5 (2.1, 17.0)	-0.41	0.68	20.8 (3.3, 38.4)	3.5 (-3.6, 10.5)	-2.03	0.05
Pre-delivery^b^	20.9 (10.9, 30.9)	21.0 (10.5, 31.4)	0.01	0.99	34.8 (13.7, 55.8)	28.6 (10.7, 46.4)	-0.46	0.64
Post-delivery^c^	13.1 (4.4, 21.8)	8.5 (1.2, 15.8)	-0.82	0.42	20.8 (3.3, 38.4)	6.9 (-2.9, 16.7)	-1.43	0.16
Change from pre- to post-delivery	-8.3 (-20.3, 3.6)	-10.3 (-23.1, 2.4)	-0.23	0.82	-13.0 (-36.7, 10.7)	-21.7 (-43.7, 0.60)	-0.53	0.60
**≥ 80% Adherence**, % (95% CI)								
Final 30 days of intervention	30.9 (18.3, 43.5)	29.1 (16.7, 41.5)	-0.21	0.84	50.0 (28.4, 71.6)	34.5 (16.1, 52.9)	-1.13	0.26
Full Intervention^a^	36.8 (25.0, 48.5)	34.9 (22.8, 47.0)	-0.22	0.83	66.7 (46.3, 87.0)	41.4 (22.3, 60.5)	-1.87	0.07
Pre-delivery^b^	55.2 (43.0, 67.4)	50.0 (37.2, 62.8)	-0.59	0.56	78.3 (60.0, 96.5)	60.7 (41.4, 80.0)	-1.36	0.19
Post-delivery^c^	36.1 (23.7, 48.5)	33.9 (21.5, 46.3)	-0.25	0.81	58.3 (37.1, 79.6)	37.9 (19.2, 56.7)	-1.48	0.14
Change from pre- to post-delivery	-23.3 (-35.3, -11.3)	-17.2 (-30.4, -4.1)	0.68	0.49	-21.7 (-40.0, -3.5)	-25.0 (-42.1, -7.9)	-0.27	0.79
**Mean adherence**, % (95% CI)								
Final 30 days of intervention	49.6 (39.4, 59.8)	52.9 (43.3, 62.6)	0.48	0.63	63.4 (48.3, 78.6)	63.1 (50.8, 75.5)	-0.03	0.97
Full Intervention^a^	63.4 (56.2, 70.5)	62.1 (54.5, 69.7)	-0.23	0.82	75.0 (62.7, 87.3)	66.3 (56.2, 76.3)	-1.14	0.26
Pre-delivery^b^	70.2 (62.7, 77.7)	68.5 (60.8, 76.1)	-0.33	0.75	83.2 (72.0, 94.5)	73.8 (63.0, 84.7)	-1.24	0.23
Post-delivery^c^	56.7 (48.2, 65.3)	54.4 (45.7, 63.2)	-0.38	0.71	67.8 (53.7, 81.8)	59.3 (47.7, 70.9)	-0.96	0.34
Change from pre- to post-delivery	-15.9 (-22.7, -9.2)	-14.3 (-21.6, 7.0)	0.33	0.74	-16.2 (-25.7, 6.8)	-15.5 (-27.5, -3.6)	0.10	0.93
**HIV disease progression**								
Mean CD4-cell count at PPM3, cells/µL (SD)	747.0 (443.7)	877.8 (606.0)	1.40	0.16	672.5 (370.5)	911.8 (592.1)	1.79	0.08
Change in CD4-cell count, baseline to PPM3								
Mean change, cells/µL (SD)	-215.7 (424.1)	-291.5 (483.4)	-0.95	0.35	-104.8 (306.7)	-362.6 (509.9)	-2.27	0.03
Proportion whose CD4-cell count rose, n (%)	15 (22.1)	8 (12.7)	-1.46	0.15	9 (37.5)	5 (17.2)	-1.64	0.11
Undetectable viral load at PPM3, n (%)	39 (57.4)	27 (42.9)	-1.66	0.10	14 (58.3)	17 (58.6)	0.02	0.98

^a^ Numbers for full intervention were: ITT = 68 (intervention), 63 (comparison); PP = 24 (intervention), 29 (comparison)

^b^ Two women delivered prior to the start of the intervention and were excluded from the pre-delivery period outcomes. ITT numbers for pre-delivery period were 67 (intervention), 62 (comparison)

^c^ Eleven women had no adherence data for the post-delivery period. ITT numbers for post-delivery period were 61 (intervention), 59 (comparison)

#### ≥80% adherence and additional secondary outcomes

Less than one-third in both groups achieved ≥80% adherence (30.9% vs. 29.1%, t = − 0.021, p = 0.84) in the final 30 days. Over the full intervention, ≥80% adherence was higher in both groups, and highest among intervention participants (36.8% vs. 34.9%, t = -0.22, p = 0.83), but not significantly so. Pre-delivery, most participants achieved the ≥80% threshold, with a higher proportion of intervention participants reaching this level (55.2% vs. 50.0%, t = -0.59, p *=* 0.56). Post-delivery, ≥80% adherence fell to 36.1% and 33.9% in intervention vs. comparison groups, respectively (t = -0.25, p *=* 0.81). Mean adherence in both groups was low, ranging from 50 to 70% over time, and showed patterns similar to threshold measures: adherence was higher in the intervention group in all periods, but not significantly, and declined significantly in the post-delivery period (Table 2; Fig. 1).

### Effect of real-time feedback on adherence outcomes: PP analysis

#### ≥95% adherence

The PP cohort demonstrated higher adherence than the full sample, particularly in intervention participants (Table 2). In the final 30 days, 16.7% vs. 10.3% (t = -0.67, p *=* 0.51) of intervention vs. comparison participants achieved ≥ 95% adherence. Over the full intervention, ≥95% adherence was achieved by 20.8% vs. 3.5% of intervention vs. comparison groups, respectively (t = -2.03, p *=* 0.05). Approximately one-third of participants achieved ≥95% adherence (34.8% vs. 28.6%, t = -0.46, p = 0.64) among intervention and comparison participants, respectively, pre-delivery, but these proportions again declined sharply post-delivery to 20.8% and 6.9% (t = -1.43, p = 0.16).

#### ≥80% adherence and additional secondary outcomes

Secondary analyses revealed consistently higher adherence in the PP cohort compared to the full sample. As with the ≥95% threshold, adherence was higher in intervention vs. comparison participants, though not significantly so, in part due to small numbers (Table 2). In the final 30 days, 50.0% vs. 34.5% (t = -1.13, p = 0.26) of intervention vs. comparison groups, respectively, achieved ≥80% adherence. These proportions rose during the intervention (66.7% vs. 41.4%, t = -1.87, p = 0.07) and were highest in the pre-delivery period (78.3% vs. 60.7%, t = -1.36, p = 0.19), before declining post-delivery in both groups (58.3% vs. 37.9%, t = -1.48, p = 0.14). Mean adherence was consistently higher than in the full sample, ranging from 59.3% (in comparison participants, in the post-delivery period) to 83.2% (in intervention participants, in the pre-delivery period) and exhibited similar patterns as other measures: higher adherence in the intervention group, but without reaching significance, and notable declines from pre- to post-delivery periods (see Table 2).

#### Monthly adherence over time and adherence patterns

**Fig. 2:**
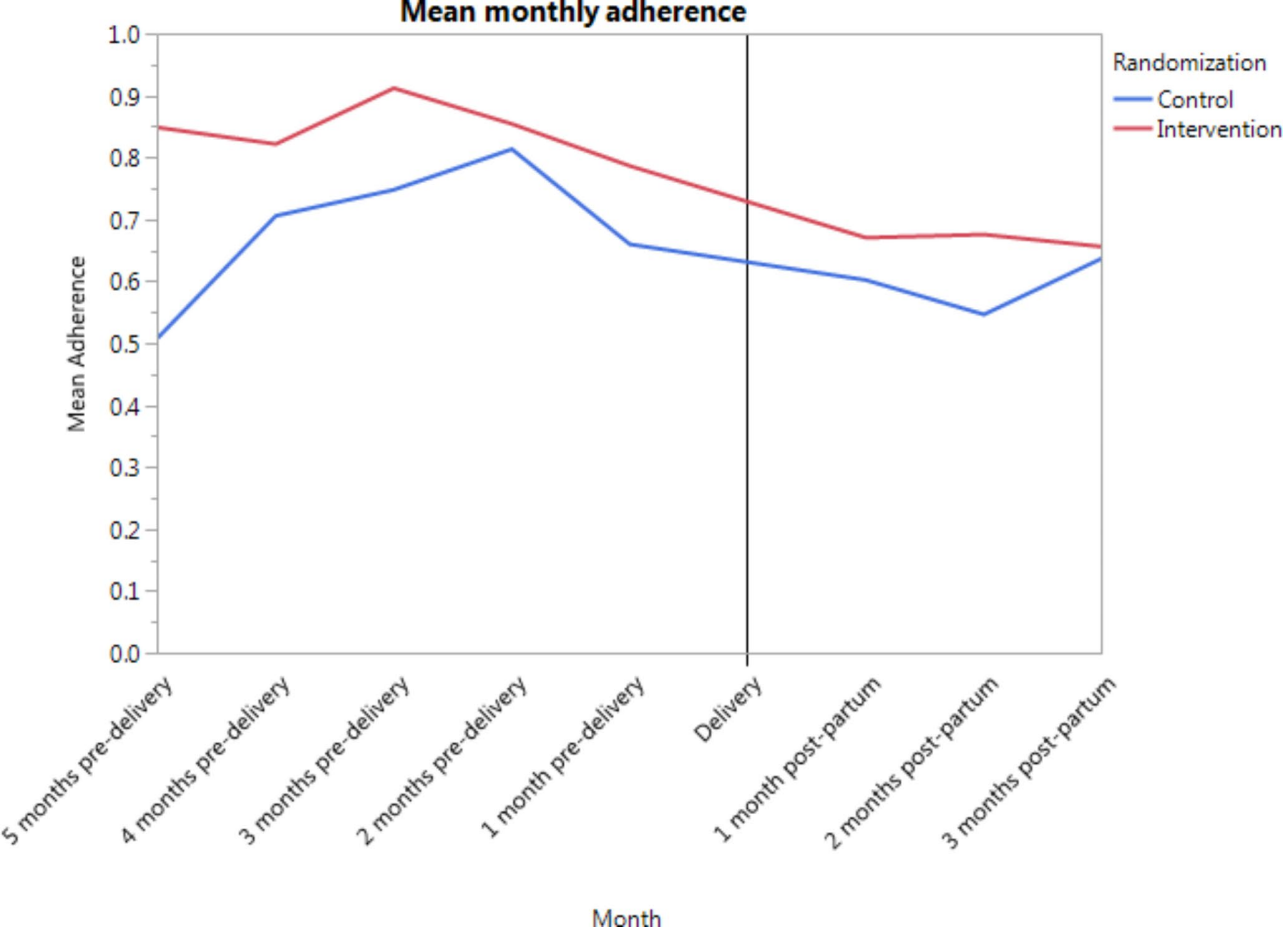
Mean monthly adherence, Per Protocol Group (n = 51)^a^ (^a^Two women delivered prior to the start of the intervention. Because they had no pre-delivery outcomes, they were excluded from this analysis)

**Fig. 3:**
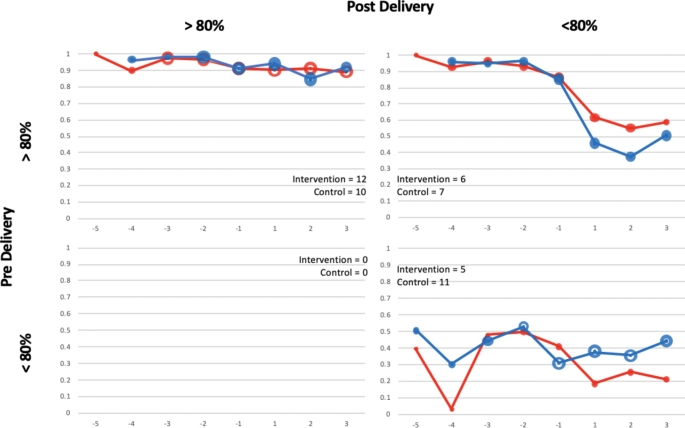
Adherence Behavior Trends, Per Protocol Group (n = 51)^a^ (^a^Two women delivered prior to the start of the intervention. Because they had no pre-delivery outcomes, they were excluded from this analysis)

As shown in Fig. 2, intervention participants in the PP cohort had consistently higher monthly adherence over the intervention than their comparison counterparts. Three distinctive pre- and post-delivery patterns emerged: 43.1% of participants had high pre- and high post-delivery adherence, 25.5% had high pre-delivery and low post-delivery adherence, and 31.4% had low adherence both pre- and post-delivery (Fig. 3). A similar proportion of intervention vs. comparison participants displayed a high/low pattern, but 52% vs. 36% had a high/high pattern, and 22% vs. 39% had a low/low pattern in intervention vs. comparison groups, respectively.

### Measures of HIV disease progression

The ITT analyses of disease progression indicated no positive intervention impact (Table 2). Mean CD4-cell count was higher at PPM3 in the comparison vs. the intervention group (877.8 vs. 747.9 cells/µL) but not significantly (t = 1.40, p = 0.16). In both groups, mean CD4-cell count declined from baseline to PPM3; the decline was sharper in comparison participants (291.5 vs. 215.7 cells/µL, but did not achieve significance; t = -0.95, p = 0.35). Less than one-fourth in both groups (22.1% and 12.7%, respectively; t = -1.46, p = 0.15) experienced an increase in CD4-cell count over the intervention. A higher proportion of intervention participants achieved viral suppression at PPM3 (57.4% vs. 42.9%), but the difference was not significant (t = -1.66, p = 0.10).

The PP analysis yielded similar patterns, though differences were greater in CD4-cell counts at PPM3 and in proportions whose CD4-cell count increased over the intervention (neither was significant) (Table 2). The mean decrease in CD4-cell count was less in the intervention group (-104.8 vs. -362.6), and significant (t = 2.27, p = 0.03), indicating potential benefit from the intervention in this cohort. At PPM3, a similar proportion (58.3% and 58.6% in intervention and comparison participants, respectively) had an undetectable viral load (t = 0.02, p = 0.98).

#### Treatment interruptions

The intervention group had fewer treatment interruptions (TIs) > 72 h in both the adjusted and unadjusted models of the ITT analysis (Table 3). In the adjusted model, the intervention group had a 46% mean overall reduction of TIs (IRR = 0.54, chi-sq = 4.49, p = 0.03). There was no significant difference between groups in TIs pre-delivery, but a significant reduction in the intervention group (46%) post-delivery in the adjusted model (IRR = 0.54, chi-sq = 4.50, p = 0.03).

**Table 3:** Poisson Regression Model for Count of TIs (> 72 h) in intervention vs. comparison groups for full intervention, pre-delivery, post-delivery periods

		Intention to treat (n = 131)						Per protocol (n = 53)					
**Time Period**	**Outcome**	**# TIs** **(I, C)**^**b**^	**IRR**	**SE**	***Z/chi-sq test***	***p***	**95% CI**	**# TIs** **(I, C)**^**b**^	**IRR**	**SE**	***Z/chi-sq test***	***p***	**95% CI**
Full intervention	Unadjusted Count of TI	(113, 160)	0.72	0.26	-1.29	0.20	(0.43, 1.19)	(35, 75)	0.49	0.57	-1.26	0.21	(0.16, 1.48)
	Adjusted Count of TI^a^		0.54	0.16	4.49	0.03	(0.30, 0.95)		0.23	0.12	7.75	0.0054	(0.08, 0.65)
Pre-delivery	Unadjusted Count of TI	(59, 77)	0.91	0.38	-0.25	0.80	(0.43, 1.91)	(10, 35)	0.46	0.93	-0.84	0.40	(0.07, 2.84)
	Adjusted Count of TI^a^		0.68	0.31	0.72	0.40	(0.27, 1.67)		0.05	0.03	31.54	< 0.0001	(0.02, 0.15)
Post-delivery	Unadjusted Count of TI	(56, 88)	0.67	0.27	-1.49	0.14	(0.40, 1.13)	(23, 44)	0.51	0.48	-1.44	0.15	(0.20, 1.28)
	Adjusted Count of TI^a^		0.54	0.16	4.50	0.03	(0.30, 0.95)		0.41	0.22	2.73	0.10	(0.15, 1.18)

^a^Model adjusted by maternal age, education, gestational age, parity, marital status, and study site

^b^(I, C) = (Intervention, Comparison)

Similar patterns were observed in the PP analysis. In the adjusted model, the intervention group experienced a 77% reduction in TIs (IRR = 0.23, chi-sq = 7.75, p = 0.005), with significant differences between groups both pre- and post-delivery. Pre-delivery, the intervention group experienced a significant reduction in TIs in the adjusted model (95%) (IRR = 0.05, chi-sq = 31.54, p < 0.0001). Post-delivery, the occurrence of TIs was lower (49% and 59%) in the intervention group in the unadjusted (IRR = 0.51, chi-sq = -1.44, p = 0.15) and adjusted (IRR = 0.41, chi-sq = 2.73, p = 0.10) models, but not statistically significant.

## Discussion

In this randomized intervention trial, we assessed the impact of real-time text message reminders and data-informed counseling on adherence to ART among PPWLH in Uganda. Our prior investigation of this approach in PLWH in China, with successful results [20, 39, 40], encouraged the hypothesis that use of real-time reminders might prove to be effective in promoting adherence behaviors elsewhere. The WiseMama trial, designed to test this hypothesis, had notable strengths: it was implemented among PPWLH, a population known to struggle with both retention in care and ART adherence and in need of extra support; it made use of powerful technology that many researchers hope can be harnessed to address challenges among struggling HIV populations; it included a lead-in period of one month to ensure that prospective trial participants could use the WPM as designed; and it utilized multiple measures for the primary adherence outcome as well as several secondary measures in order to provide a rigorous, multi-faceted assessment of impact. While our ITT analyses failed to show significant effect on adherence behaviors in our study population, our findings highlight several important results, including: persistently low adherence generally among PPWLH, with significant declines in adherence measures post-delivery; a suggestion of positive impact on adherence in a sub-sample of the study population who completed the intervention and were able to use the monitoring device as designed; a significant reduction in TIs in the same sub-sample; and several different behavioral adherence patterns among our study participants, all of which underscore the diverse experiences of this population and the need for a range of different interventions to support them. Together with the results in a companion article describing the intervention’s impact on ART retention [35], these findings highlight the challenge of attaining the UNAIDS 95-95-95 goals and the importance of continued research to identify effective measures to help maximize the full benefits of Option B + for pregnant and postpartum women [41,42].

First, given the potential the intervention held for impact, what might explain its general failure? Our measure of adherence may have underestimated actual dose-taking among study participants as well as intervention impact. As described in the methods section, days with no recorded WPM openings, accompanied by indication from the WPM central server of a dead battery, were excluded from adherence calculations. It is possible that participants were more adherent during these periods than our measures indicate because the WPM devices were unable to document such behavior accurately. Having a functioning device was critical not just to measuring adherence but also to implementation of the intervention, as it was the mechanism by which the WPM’s server triggered reminders to participants’ cell phones when doses were missed. We believe this explanation is unlikely given the low levels of viral suppression in both groups at the end of the intervention, which supports our finding of poor adherence generally and lack of intervention effect. As we have noted previously, our study participants experienced unexpected challenges keeping their WPM batteries charged [17,35,43,44]. While our design specified a one-month lead-in period prior to randomization for the express purpose of confirming the capacity to use the WPM as required for the trial, this plan did not adequately account for the difficulties participants encountered trying to keep the WPM devices fully charged over a longer timeframe. As reported in the results, we found a high degree of missing adherence data. Since the levels were similar between groups, we do not believe it affects our main findings.

Perhaps more importantly, a number of structural barriers to effective medication management may have presented challenges too difficult to overcome with simple text message reminders. Past studies, including our team’s own qualitative data [45,46] have documented food shortages [26,46,47], the cost and logistical challenge of traveling to the clinics [26,31,47,48], and fear of stigma following inadvertent disclosure of HIV status [49–52] as barriers to adherence. These issues highlight the reality that, while Option B + is well-intentioned, many pregnant and postpartum women lack the support to maintain high adherence to ART. If they did have strong support, triggered text messages and data-informed counseling might provide additional encouragement; without it, this type of intervention is likely insufficient. This point is underscored by the challenges participants faced completing scheduled hospital visits to collect ART medication [17]. As described above, the study team called participants who missed a scheduled hospital visit. This was done purposefully to support study fidelity, but it may also have diluted the potential effect of the intervention inadvertently. After taking these possible explanations into account, we posit that a combination of technical and other interpersonal, social, and structural barriers to using the WPM as intended and being able to respond to text reminders when they were sent hindered the ability of the intervention to serve as an effective adherence support tool in our study population.

Yet, while the ITT analysis of adherence outcomes indicated no significant intervention impact across the study population, other analyses tell a slightly different story. First, the PP analysis suggests that the intervention may, in fact, work for some PPWLH. No obvious characteristics (age, education, marital status) explained the generally higher adherence, as well as lower decline in CD4-cell count, in the PP cohort compared to the full sample. However, the fact that this group of participants, by definition, remained in the study until its conclusion and were relatively more able to keep their device batteries charged suggests they may have been more motivated or determined to adhere to their ART regimens. Because this participant cohort, by virtue of using fully functional devices that could deliver the intervention properly, was best equipped to benefit from the reminders, it is encouraging to see signs of a difference in adherence outcomes over time. Given the small sample included in this analysis, further research is needed for conclusive evidence.

Separate indications of potential intervention benefit were in the outcomes on TIs, which were significant across the study population. The evidence suggests that TIs are critical in drug resistance [11], so an impact on TIs may be as important, or even more so, than an effect on mean adherence. Previous work has found that patients who keep their cumulative adherence above 95% may not be able to recover from a single TI of more than 28 days and proceed to fail treatment [53]. Treatment interrupters may be generally good at taking treatment but experience an event (such as moving or childbirth) that results in a TI [53]. An RCT using WPM-triggered reminders in South Africa showed no effect on overall adherence, but observed fewer sustained TIs [21]. In our study population, we similarly observed significantly fewer TIs among intervention participants in both the ITT and PP analyses. It is conceivable that the motivation to be adherent to ART while pregnant to protect the baby from mother-to-child transmission of HIV, in combination with the WPM reminders and data-informed counseling, played a role in reducing TIs pre-delivery. As previously documented in the literature, we hypothesize that participants’ medication-taking behaviors changed post-delivery due to a complex set of interpersonal and structural factors [54–58]. The need to explore what interventions would effectively motivate and support HIV-positive women to continue taking ART post-delivery remains critical for this population [54].

Finally, we observed unique ART adherence patterns among the PP cohort, which add useful insight into the medication-taking behaviors of PPWLH. Most obviously, these patterns underscore the fact that adherence behaviors are not equal across all PPWLH. Conceptualizing behaviors as revealing “high” or “low” average adherence, we found three distinctive ART adherence patterns in the PP cohort as participants transitioned through pregnancy and into their post-delivery lives. The most dominant pattern, characterizing nearly half our study population, was a high/high pattern in pre- and post-delivery periods. Notably, this pattern was more typical of intervention than comparison participants (52% vs. 36%, respectively). At the other extreme, nearly one-third were consistently low in both periods, a pattern more prominent among the comparison group (22% vs. 39%). About one-quarter of both groups displayed a major change from “high” to “low” adherence behaviors between the pregnancy and postpartum periods. We thus highlight the heterogeneity of experience and constraints that must be matched with different interventions for effective support. In addition, we found somewhat better overall adherence in the rural study site as compared to the urban site, similar to the outcomes (previously reported) related to retention in care [17]. Anecdotally, study collaborators believed that Entebbe-based participants may have been more mobile because many were partners of military personnel and returned to their home villages to deliver, which challenged their ability to remain in care in Entebbe and to follow study procedures post-delivery. While we were unable to confirm this theory, our findings highlight the importance of understanding local migration patterns and their impact on access to care.

We acknowledge several study limitations. First, we were unable to blind participants and clinicians, who provided data-informed counseling to intervention participants. We do not believe bias in counseling (for one group or the other) is of major concern due to the comprehensive counseling training provided to all clinicians, as well as the null finding on intervention effect. Second, technical challenges on the part of the study population through the life of the study, particularly related to keeping the WPM devices charged, were substantial and unanticipated given the pre-randomization one-month lead-in period. Third, the intervention was implemented for only three months postpartum; a timeframe of one year or more after delivery might show a less sharp decline postpartum compared to pre-delivery as women regain a more regular schedule post-delivery. Fourth, the study sample was not large, and our statistical power was limited to observe relatively small but possibly still meaningful differences. Fifth, it is not possible to tease out impact between the text messages and counseling, as both activities were intervention features. Lastly, the findings of this intervention may not be generalizable to other settings as they are dependent on culture- and location-specific behaviors that influence adherence to ART.

## Conclusions

This study contributes useful findings about ART adherence behaviors of PPWLH in a low-resource setting in sub-Saharan Africa. We observed generally low ART adherence in our study population, along with significant declines in adherence between pre- and post-delivery. Our main analyses did not confirm a positive intervention effect, though supplemental analyses hinted at potential benefit for some PPWLH. We conclude that this type of technology-based intervention may not be appropriate for PPWLH generally, but may be effective for certain sub-groups among this population and in settings where cell coverage and electricity are more reliable. Given the mixed evidence from a variety of locations, and the continued need for adherence interventions for vulnerable populations, we recommend further investigation into real-time SMS reminders and data-informed counseling in other locations and populations, targeting those populations most likely to benefit.

## Electronic Supplementary Material

Below is the link to the electronic supplementary material.

Supplementary Material 1

Supplementary Material 2

## Data Availability

We are continuing to analyze data in preparation for more manuscripts. Once we complete all analyses, we plan to make the data available in a publicly-accessible repository.

## Code Availability

The codes for the statistical analyses are not available publicly at this time. Once all analyses are completed, we will consider requests for the codes from interested researchers.

## Declarations

### Data Transparency

A subset of study findings was presented to government officials and non-government personnel in Uganda in Feb-March 2018 and at the 12th International Conference on HIV Treatment and Prevention (June 2017). However, all findings presented previously were preliminary and did not include the full set of analyses presented here.
