# Supplementary material for: Real-time Feedback to Improve HIV Treatment Adherence in Pregnant and Postpartum Women in Uganda: A Randomized Controlled Trial
Source: AIDS Behav. 2022 Jun 15;26(12):3834–47. doi: 10.1007/s10461-022-03712-7 (PMC9640413; doi:10.1007/s10461-022-03712-7)
Supplement: Supplementary file 1 — Supplementary Material 1 [file 10461_2022_3712_MOESM1_ESM.docx]

**Supplemental Table 1a.** **ITT Analysis - Mityana**

Mean adherence and proportion of women reaching >95% and >80% thresholds for full intervention, pre-delivery, post-delivery and last 30 day periods in Mityana

| **Time Period** | **Outcome** | **Intervention Group**  n=33 | | **Comparison Group**  n=33 | | **Overall**  n=66 | | ***t-test*** | ***P*** |
| --- | --- | --- | --- | --- | --- | --- | --- | --- | --- |
|  |  | n | % (95% CI) | n | % (95% CI) | n | % (95% CI) |  |  |
| Full intervention | Mean adherence | 33 | 63.4 (52.9, 73.8) | 33 | 64.9 (55.6, 74.3) | 66 | 64.1 (57.3, 71.0) | 0.23 | 0.82 |
|  | >95% adherence | 4 | 12.1 (0.4, 23.9) | 2 | 6.1 (-2.5, 14.7) | 6 | 9.1 (2.0, 16.2) | -0.85 | 0.40 |
|  | >80% adherence | 13 | 39.4 (21.8, 57.0) | 11 | 33.3 (16.4, 50.3) | 24 | 36.4 (24.4, 48.3) | -0.50 | 0.62 |
| Pre-delivery^a^ | Mean adherence | 32 | 70.8 (59.4, 82.1) | 33 | 71.7 (61.9, 81.4) | 65 | 71.2 (64.0, 78.5) | 0.12 | 0.91 |
|  | >95% adherence | 7 | 21.9 (6.7, 37.0) | 4 | 12.1 (0.4, 23.9) | 11 | 16.9 (7.6, 26.3) | -1.04 | 0.30 |
|  | >80% adherence | 19 | 59.4 (41.4, 77.4) | 18 | 54.5 (36.6, 72.5) | 37 | 56.9 (44.6, 69.3) | -0.39 | 0.70 |
| Post-delivery^b^ | Mean adherence | 29 | 55.1 (42.4, 67.7) | 33 | 57.1 (46.0, 68.1) | 62 | 56.1 (48.0, 64.2) | 0.24 | 0.81 |
|  | >95% adherence | 3 | 10.3 (-1.4, 22.1) | 2 | 6.1 (-2.5, 14.7) | 5 | 8.1 (1.1, 15.0) | -0.60 | 0.55 |
|  | >80% adherence | 9 | 31.0 (13.1, 48.9) | 11 | 33.3 (16.4, 50.3) | 20 | 32.3 (20.3, 44.2) | 0.19 | 0.85 |
| Last 30 days of intervention^c^ | Mean adherence | 26 | 49.7 (35.1, 64.4) | 29 | 55.5 (44.0, 67.0) | 55 | 52.8 (43.9, 61.7) | 0.64 | 0.53 |
|  | >95% adherence | 3 | 11.5 (-1.6, 24.7) | 1 | 3.4 (-3.6, 10.5) | 4 | 7.3 (0.2, 14.4) | -1.11 | 0.27 |
|  | >80% adherence | 7 | 26.9 (8.7, 45.2) | 6 | 20.7 (5.0, 36.4) | 13 | 23.6 (12.0, 35.2) | -0.53 | 0.60 |

**^a^** One woman delivered prior to the start of the intervention and was excluded from the pre-delivery period outcomes.

^b^ Four women had no adherence data for the post-delivery period.

^c^ 11 women had no adherence data for the last 30 days of the intervention period.
