# Supplementary material for: Real-time Feedback to Improve HIV Treatment Adherence in Pregnant and Postpartum Women in Uganda: A Randomized Controlled Trial
Source: AIDS Behav. 2022 Jun 15;26(12):3834–47. doi: 10.1007/s10461-022-03712-7 (PMC9640413; doi:10.1007/s10461-022-03712-7)
Supplement: Supplementary file 2 — Supplementary Material 2 [file 10461_2022_3712_MOESM2_ESM.docx]

**Supplemental Table 1b. ITT Analysis – Entebbe**

Mean adherence and proportion of women reaching >95% and >80% thresholds for full intervention, pre-delivery, post-delivery and last 30 day periods in Entebbe

| **Time Period** | **Outcome** | **Intervention Group**  n=35 | | **Comparison Group**  n=30 | | **Overall**  n=65 | | ***t-test*** | ***P*** |
| --- | --- | --- | --- | --- | --- | --- | --- | --- | --- |
|  |  | n | % (95% CI) | n | % (95% CI) | n | % (95% CI) |  |  |
| Full intervention | Mean adherence | 35 | 63.4 (52.9, 73.8) | 30 | 59.1 (46.4, 71.8) | 65 | 61.4 (53.5, 69.3) | -0.53 | 0.59 |
|  | >95% adherence | 4 | 11.4 (0.3, 22.5) | 4 | 13.3 (0.4, 26.2) | 8 | 12.3 (4.1, 20.5) | 0.23 | 0.82 |
|  | >80% adherence | 12 | 34.3 (17.7, 50.8) | 11 | 36.7 (18.4, 55.0) | 23 | 35.4 (23.4, 47.3) | 0.20 | 0.84 |
| Pre-delivery^a^ | Mean adherence | 35 | 69.7 (59.2, 80.2) | 29 | 64.9 (52.3, 77.4) | 64 | 67.5 (59.6, 75.4) | -0.61 | 0.54 |
|  | >95% adherence | 7 | 20.0 (6.1, 33.9) | 9 | 31.0 (13.1, 48.9) | 16 | 25.0 (14.1, 35.9) | 0.99 | 0.32 |
|  | >80% adherence | 18 | 51.4 (34.0, 68.8) | 13 | 44.8 (25.6, 64.1) | 31 | 48.4 (35.9, 61.0) | -0.52 | 0.61 |
| Post-delivery^b^ | Mean adherence | 32 | 58.2 (46.0, 70.5) | 26 | 51.1 (36.2, 66.0) | 58 | 55.0 (45.8, 64.3) | -0.76 | 0.45 |
|  | >95% adherence | 5 | 15.6 (2.3, 28.9) | 3 | 11.5 (-1.6, 24.7) | 8 | 13.8 (4.6, 22.9) | -0.45 | 0.66 |
|  | >80% adherence | 13 | 40.6 (22.6, 58.6) | 9 | 34.6 (15.0, 54.2) | 22 | 37.9 (25.1, 50.8) | -0.46 | 0.65 |
| Last 30 days^c^ | Mean adherence | 29 | 49.4 (34.3, 64.5) | 26 | 50.1 (33.4, 66.8) | 55 | 49.7 (38.9, 60.6) | 0.06 | 0.96 |
|  | >95% adherence | 6 | 20.7 (5.0, 36.4) | 4 | 15.4 (0.5, 30.2) | 10 | 18.2 (7.7, 28.7) | -0.50 | 0.62 |
|  | >80% adherence | 10 | 34.5 (16.1, 52.9) | 10 | 38.5 (18.4, 58.5) | 20 | 36.4 (23.2, 49.5) | 0.30 | 0.76 |

**^a^** One woman delivered prior to the start of the intervention and was excluded from the pre-delivery period outcomes.

^b^ Seven women had no adherence data for the post-delivery period.

^c^ Ten women had no adherence data for the last 30 days of the intervention period.
